# *Cirsium japonicum* var. *Maackii* Improves Cognitive Impairment under Amyloid Beta_25-35_-Induced Alzheimer's Disease Model

**DOI:** 10.1155/2022/4513998

**Published:** 2022-01-07

**Authors:** Qi Qi Pang, Ji-Hyun Kim, Ji Myung Choi, Jia-Le Song, Sanghyun Lee, Eun Ju Cho

**Affiliations:** ^1^Department of Food Science and Nutrition, Institute of Kimchi Research, Pusan National University, Busan 46241, Republic of Korea; ^2^Department of Nutrition and Food Hygiene, School of Public Health, Guilin Medical University, Guilin 541004, China; ^3^Department of Plant Science and Technology, Chung-Ang University, Anseong 17546, Republic of Korea

## Abstract

Abnormal production and degradation of amyloid beta (A*β*) in the brain lead to oxidative stress and cognitive impairment in Alzheimer's disease (AD). *Cirsium japonicum* var. *maackii* (CJM) is widely used as an herbal medicine and has antibacterial and anti-inflammatory properties. This study focused on the protective effect of the ethyl acetate fraction from CJM (ECJM) on A*β*_25-35_-induced control mice. In the T-maze and novel object recognition test, ECJM provided higher spatial memory and object recognition compared to A*β*_25-35_ treatment alone. In the Morris water maze test, ECJM-administered mice showed greater learning and memory abilities than A*β*_25-35_-induced control mice. Additionally, ECJM-administered mice experienced inhibited lipid peroxidation and nitric oxide production in a dose-dependent manner. The present study indicates that ECJM improves cognitive impairment by inhibiting oxidative stress in A*β*_25-35_-induced mice. Therefore, CJM may be useful for the treatment of AD and may be a potential material for functional foods.

## 1. Introduction

Alzheimer's disease (AD) is a neurodegenerative disease, and its main feature is the excessive accumulation of amyloid beta (A*β*) peptides in the brain [1]. A*β* is derived from amyloid precursor protein (APP) through the proteolytic cleavage of a family of enzymes (*β*- and *γ*-secretase), which leads to memory loss and cognitive decline by the formation of senile plaques [2]. Previous studies have demonstrated that the accumulation and aggregation of A*β* (i.e., oligomers and senile plaques) are highly neurotoxic and form A*β* deposit/fibrils, which can contribute to AD development [3]. A*β* oligomers in the early stage and A*β* plaques in later stages are established, so they are observed in the AD brain for decades before the onset of memory/behavior/cognitive symptoms [4]. Therefore, therapeutic approaches targeting A*β* by regulating the A*β* mechanism have attracted attention. The A*β*_25-35_ fragment used in the present study, a core toxic fragment cleaved from the full-length A*β* peptide, plays a critical role in the development of AD by inducing oxidative stress, neurotoxicity, and inflammatory response [5].

Oxidative stress is caused by the disequilibrium of the redox reaction and generates excessive reactive oxygen species (ROS) in the body [6]. The brain, which consumes large amounts of oxygen, may be notably sensitive to oxidative damage [7]. Moreover, several studies have reported that metal ions (e.g., zinc, iron, and copper) that bind A*β* exist in A*β* plaques and modulate the aggregation process. A*β* plaques with entrapped metal ions produce free radicals, such as ROS and reactive nitrogen species (RNS) [8]. Thus, the accumulation of A*β* induces oxidative stress in the brain, resulting in lipid peroxidation, protein oxidation, and cognitive impairment [5, 9]. Moreover, many studies have reported that A*β* activates microglia, leading to the release of inflammatory cytokines, such as interleukin-1*α*, interleukin-1*β*, and tumor necrosis factor-*α* [10]. Subsequently, pathologic changes in the microglia contribute to the secretion of proinflammatory materials, including ROS and RNS, which trigger detrimental inflammation. This inflammatory-oxidative stress cycle means that A*β* induces inflammatory signals, which then release ROS and RNS. This further evokes inflammation, and the entire process develops into an inflammatory-oxidative stress cycle [11, 12]. Therefore, A*β*_25-35_-induced *in vivo* mouse models have been used to investigate oxidative stress and cognitive impairment in AD patients.

*Cirsium japonicum* var. *maackii* (CJM) is a perennial herb of the chrysanthemum family and is mainly distributed in China, Korea, Japan, and other places [13]. Several pharmacological benefits of CJM have been reported that it has anti-inflammatory and antihypertensive effects [14, 15]. In addition, Oh et al. [16] demonstrated that the CJM flower exerted anticancer activity by inhibition the PI3K-Akt signaling pathway. In addition, the CJM indicated the remarkable protective activities from oxidative stress-related diseases including diabetes mellitus. Furthermore, Wagle et al. [17] demonstrated that isolated compound luteolin from CJM showed the anti-AD activity through the suppression of BACE1 (*β*-secretase), which formed the amyloid beta by cleaving the amyloid *β* precursor protein. Flavonoids such as cirsimarin, cirsimaritin, hispidulin, and pectolinarin are usually isolated from CJM, and these mediate the pharmacological properties of CJM [18]. Lee et al. [18] verified that the highest constituent from EtOAc fraction of CJM is cirsimaritin, following cirsimarin and hispidulin. In addition, apigenin and luteolin are major constituents in the EtOAc fraction of CJM flower [19]. In particular, we reported that pectolinarin from the EtOAc fraction of CJM has neuroprotective effects [20].

Many studies have demonstrated that the ethyl acetate (EtOAc) fraction of CJM (ECJM) significantly inhibited aldose reductase, *α*-glucosidase, oxidative stress, and inflammation [14, 17, 18, 21, 22]. Moreover, in our previous study, among the four fractions of CJM, including EtOAc, dichloromethane (CHCl_3_), *n*-butanol (BuOH), and *n*-hexane, the EtOAc fraction showed the strongest biological activity against A*β*_25-35_-induced cytotoxicity in SH-SY5Y neuronal cells [20]. This suggests that CJM may have a potential protective effect on AD and ECJM is the major active fraction with beneficial health effects. Accordingly, the EtOAc fraction of CJM was selected for further *in vivo* studies to investigate its protective effects against A*β*_25-35_-induced cognitive impairment. However, there have been no studies on the protective effects of ECJM against A*β*_25-35_-induced cognitive impairment in an *in vivo* mouse model. Therefore, in the present study, we investigated the protective effects and mechanisms of ECJM on A*β*_25-35_-induced cognitive impairment in mice by exploring behavioral tests and measuring lipid peroxidation and nitric oxide (NO) production.

## 2. Materials and Methods

### 2.1. Plant Materials and Sample Preparation

The aerial part of CJM was obtained from Imsil Herbal Medicine (Imsil, Korea). Dried CJM (5.7 kg) was extracted with ethyl alcohol (EtOH) for 3 h at 65–75°C under reflux, and 667.2 g of EtOH extract was obtained (yield 11.7%). The EtOH extract was supplemented with water and partitioned with EtOAc, CHCl_3_, *n*-BuOH, and *n*-hexane in the present study; only the EtOH fraction was used to investigate the improvement effects on A*β*_25-35_-induced cognitive impairment. The EtOAc fraction (67.6 g) was obtained (yield 10.1%) and dissolved in distilled water (DW) for use [18].

### 2.2. Reagents

A*β*_25-35_, donepezil, malondialdehyde (MDA), and Griess reagent were purchased from Sigma-Aldrich Co. (St. Louis, MO, USA). Phosphoric acid and butanol were obtained from Samchun Chemical Co., Ltd. (Seoul, Korea). Thiobarbituric acid (TBA) was obtained from Lancaster Synthesis (Ward Hill, MA, USA), and trichloroacetic acid (TCA) was acquired from Kanto Chemical Co., Inc. (Tokyo, Japan).

### 2.3. Animals and Experimental Designs

**Figure 1:**
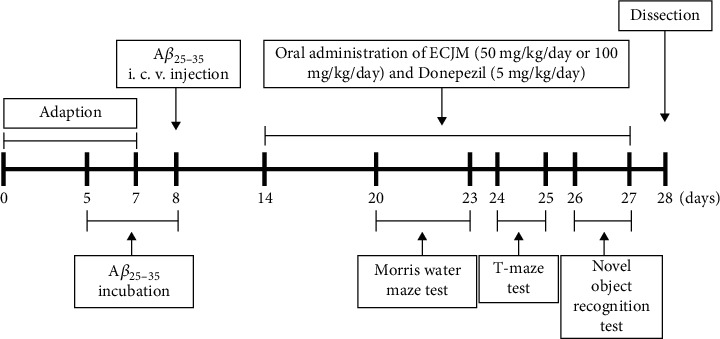
Timeline of experiments.

Male ICR mice (5 weeks old; Orient Inc., Gyeonggi-do, Korea), weighing 24–27 g, were fed in transparent plastic cages and provided with diet and water for free use. The housing environment was maintained at a temperature of 20 ± 2°C, humidity of 50 ± 10%, and light and dark cycle movements were controlled for 12 h. The mice were adapted for a 7-day period before A*β*_25-35_ administration, and a total of 35 mice were randomly divided into five groups (*n* = 7 per group) by similar average weight without significant body weight differences among groups. On the 8th day of the experiment, mice were administered 5 *μ*L A*β*_25-35_ at a concentration of 5 nM/*μ*L. Between the 14th day and 27th day of the experiment, the ECJM50 group and ECJM100 group were orally administered 50 mg/kg ECJM and 100 mg/kg ECJM, respectively, once a day for 14 days. Each group was defined as follows: (1) sham group—injection of 0.9% NaCl and oral administration of water; (2) control group—injection of A*β*_25-35_ and oral administration of water; (3) ECJM50 group—injection of A*β*_25-35_ and oral administration of ECJM (50 mg/kg/day); (4) ECJM100 group—injection of A*β*_25-35_ and oral administration of ECJM (100 mg/kg/day); and (5) donepezil group—injection of A*β*_25-35_ and oral administration of donepezil (5 mg/kg/day). ECJM and donepezil were dissolved in DW before use. In our experiment, mice were orally administered ECJM or donepezil in a volume of 100 *μ*L for 14 days by gastric gavage. All of the studies were carried out according to the guidelines of the Pusan National University Institutional Animal (PNU-IACUC, approval number: PNU-2019-2393). The timeline of the experiments is shown in Figure 1.

### 2.4. Animal Model

A*β*_25-35_ was dissolved in 0.9% NaCl solution and incubated at 37°C for 72 h at a concentration of 5 nM/*μ*L [23]. Before the intracerebroventricular (i.c.v.) injection, a mixture of tiletamine hydrochloride and zolazepam hydrochloride was prepared and mixed with xylazine at a ratio of 3 : 2 and used to anesthetize the mice. After stereotactic coordination of the mice, 5 nM of A*β*_25-35_ was injected intracerebroventricularly into the right ventricle at a rate of 5 *μ*L/3 min (anterior and posterior, -0.8 mm; posterolateral, -1.5 mm; dorsal and ventral, -2.2 mm) [24]. Mice in the control, ECJM, and donepezil groups were injected intracerebroventricularly with A*β*_25-35_ in the brain, while mice in the sham group were injected intracerebroventricularly with 0.9% NaCl.

### 2.5. T-Maze Test

The T-maze test was performed according to the method of Montgomery [25], with modifications. We used a T-shaped labyrinth device with a left arm, right arm, and starter box. They consist of black panels (the starting and goal stick length is 50 cm, the width is 13 cm, and the height is 20 cm), and they are separated by black partitions on both sides. During the training session, the left arm was blocked in the T-maze, and the mouse was placed in the start box of the T-maze and allowed to explore for 10 min. After finishing the training session, the mice were returned to their cages. After 24 h, mice were allowed to explore both the left and right arms of the T-maze for 10 min, and then, the number of entries was counted. Spatial awareness (%) was calculated as follows: (number of left‐or right‐arm mouse entries/number of total‐arm mouse entries) × 100%.

### 2.6. Novel Object Recognition Test

The novel object recognition test was conducted based on the study of Bevins and Besheer [26]. During the training session, two identical objects (A, A′) were placed in the center of an open field box (40 × 40 × 40 cm) coated with black paint. The two objects remained at a fixed position during the experiment. Each mouse was placed in the center of the box and allowed to explore for 10 min. The number of contacts per subject was also recorded. After 24 h of training session, one of the two objects was changed to a novel object (A, B) for the test session. Each mouse was placed and allowed to contact each object for 10 min. The number of contacts with familiar (A) and novel (B) objects was recorded. The novel object cognitive function (%) was calculated as follows: (the number of contacts with the familiar or novel object/the total number of contacts with the two objects) × 100%.

### 2.7. Morris Water Maze Test

The Morris water maze test was performed by Morris [27] with slight modifications. The equipment consisted of round obstacles (80 cm in diameter and 40 cm in height) and was randomly divided into four equal parts. Each quadrant had a different poster at the center of the wall as a visual cue for navigation. The equipment was filled with invisible water, and the water temperature was maintained at 22 ± 2°C. The escape platform (8 cm in diameter) was set at the center of one of the quadrants, so that it was in a fixed position approximately 1 cm below the water surface. Mice were trained three times a day for three days. During the training period, mice were placed in the water facing the wall of the pool and allowed to swim for a maximum of 60 s to find a hidden platform. If the mouse could not find the hidden platform, it was guided to stay on the platform for 15 s. After training, the mice were returned to their cages. During the test session, the experiment was performed as described previously. In the secondary test, the platform was removed, and each mouse was allowed to explore the pool for 60 s. The mice searched for the target quadrant where the platform was placed, and then, the percentage of time spent in the target quadrant was calculated. In the final test, the water was replaced with clean water, and the time to reach the exposed platform was measured.

### 2.8. Measurement of NO Scavenging Activity

NO production in tissue was measured using the method established by Bryan and Grisham [28]. After the behavioral experiment was completed, the mice were anesthetized under CO_2_, and the brain, liver, and kidneys were immediately removed. The tissues in 0.9% NaCl were homogenized, and 150 *μ*L of tissue homogenate was mixed with 130 *μ*L of DW for the measurement of NO. Afterwards, 100 *μ*L of the mixture was added to an equal volume of Griess reagent for the reaction. The absorbance of the mixture was measured at 540 nm wavelength. The level of NO production was calculated using a standard curve for sodium nitrite.

### 2.9. Measurement of Lipid Peroxidation

Lipid peroxidation was measured using the method established by Ohkawa et al. [29]. The tissues were homogenized in 20% phosphoric acid, 46 mM TBA solution, and 920 mM TCA solution. The mixture was boiled at 100°C for 20 min and immediately placed on ice. Butanol (7 mL) was added to the mixture and centrifuged at 3,000 rpm for 10 min. The supernatant was removed to measure the absorbance at 540 nm. Lipid peroxidation levels were calculated using the MDA standard curve.

### 2.10. Statistical Analysis

All results are expressed as the mean ± standard deviation. Statistical significance was determined using one-way analysis of variance (ANOVA) followed by Duncan's multiple range test. In the T-maze and novel object recognition test, Student's *t*-test was used to compare the two conditions. Statistical significance was set at *P* < 0.05.

## 3. Results

### 3.1. Effect of ECJM on Space Perceptive Ability

**Figure 2:**
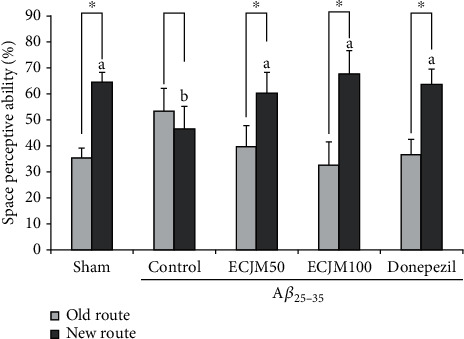
Effect of ECJM on T-maze test. (1) Sham group: injection of 0.9% NaCl and oral administration of water. (2) Control group: injection of A*β*_25-35_ and oral administration of water. (3) ECJM50 group: injection of A*β*_25-35_ and oral administration of ECJM (50 mg/kg/day). (4) ECJM100 group: injection of A*β*_25-35_ and oral administration of ECJM (100 mg/kg/day). (5) Donepezil group: injection of A*β*_25-35_ and oral administration of donepezil (5 mg/kg/day). Values are the mean ± SD. One-way ANOVA was used to compare the mean values of groups, followed by Duncan's multiple-range test post hoc. Different alphabet letters (a, b) indicate statistically significant difference (*P* < 0.05). Mean values with the same letter are not significantly different. The asterisk (∗) represents the significant difference in space perceptive abilities with old and new routes by Student's *t*-test (*P* < 0.05).

The results of the T-maze test are shown in Figure 2. The T-maze test is one of the methods used to evaluate the cognitive and memory abilities of mice in space exploration. The A*β*_25-35_-induced control group showed a 53.42% and 46.58% space exploration rate for the old and new routes, respectively. However, the space exploration of the new route was 64.61% in the sham group. The space exploration rate of the A*β*_25-35_-induced control group was significantly lower than that of the sham group. However, after oral administration of ECJM at doses of 50 and 100 mg/kg/day, the space exploration rates for the new route were 60.20% and 67.55%, respectively. The group administered donepezil as a positive control showed a new route exploration rate of 63.44%.

### 3.2. Effect of ECJM on Object Recognition Ability

**Figure 3:**
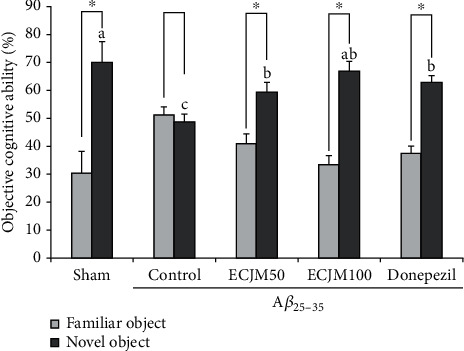
Effect of ECJM on novel objective recognition test. (1) Sham group: injection of 0.9% NaCl and oral administration of water. (2) Control group: injection of A*β*_25-35_ and oral administration of water. (3) ECJM50 group: injection of A*β*_25-35_ and oral administration of ECJM (50 mg/kg/day). (4) ECJM100 group: injection of A*β*_25-35_ and oral administration of ECJM (100 mg/kg/day). (5) Donepezil group: injection of A*β*_25-35_ and oral administration of donepezil (5 mg/kg/day). Values are the mean ± SD. One-way ANOVA was used to compare the mean values of groups, followed by Duncan's multiple-range test post hoc. Different alphabet letters (a, b, and c) indicate statistically significant difference (*P* < 0.05). Mean values with the same letter are not significantly different. The asterisk (∗) represents the significant difference in object recognition ability with familiar and novel objects by Student's *t*-test (*P* < 0.05).

During the training session, the mice did not show statistical significance for the recognition of two objects (A, A′). After 24 h, one of the objects was changed into a different object (A, B), and the results of the testing session are shown in Figure 3. The sham group of mice had a search rate of 69.79% for the novel object. Compared with the familiar object, the sham group showed more curiosity about the novel object. Conversely, the exploration of familiar and novel objects in the A*β*_25-35_-induced control group was 51.27% and 48.73%, respectively, and there was no statistically significant difference between the novel and familiar objects. Concurrently, the mice that were administered ECJM (50 or 100 mg/kg/day) and donepezil (5 mg/kg/day) displayed significantly increased cognitive ability toward novel objects. In particular, the ECJM100 group showed higher exploration of novel objects than the other groups.

### 3.3. Effect of ECJM on Learning and Memory Ability

**Figure 4:**
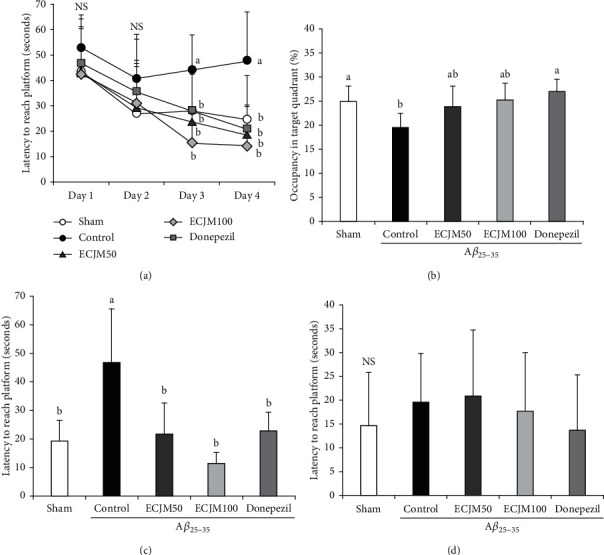
Effect of ECJM on Morris water maze test. (a) Effect of ECJM on escape latency in the Morris water maze test. (b) Effect of ECJM on occupancy time of the target quadrant in the Morris water maze test. (c) Effect of ECJM on latency to reach a hidden platform. (d) Effect of ECJM on latency to reach an exposed platform. (1) Sham group: injection of 0.9% NaCl and oral administration of water. (2) Control group: injection of A*β*_25-35_ and oral administration of water. (3) ECJM50 group: injection of A*β*_25-35_ and oral administration of ECJM (50 mg/kg/day). (4) ECJM100 group: injection of A*β*_25-35_ and oral administration of ECJM (100 mg/kg/day). (5) Donepezil group: injection of A*β*_25-35_ and oral administration of donepezil (5 mg/kg/day). Values are the mean ± SD. One-way ANOVA was used to compare the mean values of groups, followed by Duncan's multiple-range test post hoc. Different alphabet letters (a, b) indicate statistically significant difference (*P* < 0.05). Mean values with the same letter are not significantly different. NS: nonsignificance.

The Morris water maze test was used to evaluate the effect of ECJM on memory and spatial learning abilities of mice. As shown in Figure 4(a), in the A*β*_25-35_-induced control group, the time required to reach the hidden platform during the training process for 3 days was the longest among all groups. In the test session on day 4, the sham, ECJM50, ECJM100, and donepezil groups reached the hidden platform in a shorter time than the control group. The total time for each group of mice to enter the target quadrant is indicated in Figure 4(b). The A*β*_25-35_-induced control group had cognitive impairment, and the time to stay was shorter than that in the sham group. The mice in the ECJM50, ECJM100, and donepezil groups stayed for a longer time in the target quadrant compared with the control group. To investigate whether ECJM's learning and memory improvement effect on mice was related to the visual and athletic ability of mice, we also measured the latency to reach the exposed platform (Figure 4(d)). As shown in Figure 4(d), during the exposed platform test, no statistical significance was observed in any of the experimental groups, indicating that the improvement effect of ECJM on the learning and memory abilities in mice was unrelated to vision and motor ability.

### 3.4. Effect of ECJM on NO Production Induced by A*β*_25-35_

**Figure 5:**
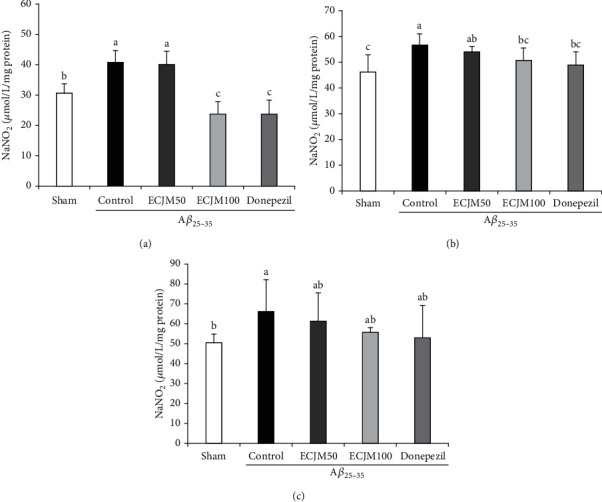
Effect of ECJM on A*β*_25-35_-induced NO production in the brain (a), liver (b), and kidney (c). (1) Sham group: injection of 0.9% NaCl and oral administration of water. (2) Control group: injection of A*β*_25-35_ and oral administration of water. (3) ECJM50 group: injection of A*β*_25-35_ and oral administration of ECJM (50 mg/kg/day). (4) ECJM100 group: injection of A*β*_25-35_ and oral administration of ECJM (100 mg/kg/day). (5) Donepezil group: injection of A*β*_25-35_ and oral administration of donepezil (5 mg/kg/day). Values are the mean ± SD. One-way ANOVA was used to compare the mean values of groups, followed by Duncan's multiple-range test post hoc. Different alphabet letters (a, b, and c) indicate statistically significant difference (*P* < 0.05). Mean values with the same letter are not significantly different.

The inhibitory effect of ECJM on NO production in the brain, liver, and kidneys is shown in Figure 5. The NO production of the sham group in the brain (Figure 5(a)) was 30.7 *μ*mol/L/mg, while that of the control group was 40.89 *μ*mol/L/mg. It could be seen that NO production significantly increased in the brain by the injection of A*β*_25-35_. Moreover, the NO production level in the ECJM50 and ECJM100 groups decreased to 40.3 *μ*mol/L/mg and 23.94 *μ*mol/L/mg, respectively, and was significantly reduced to 23.80 *μ*mol/L/mg in the donepezil group. In the liver (Figure 5(b)), the NO production level in the sham group was 46.09 *μ*mol/L/mg, while that in the control group significantly increased to 56.86 *μ*mol/L/mg. ECJM at a dose of 50 mg/kg/day showed no significant reduction of NO production, but ECJM at a dose of 100 mg/kg/day and in donepezil-administered groups reduced NO production to 50.67 *μ*mol/L/mg and 49.00 *μ*mol/L/mg, respectively. In the kidney (Figure 5(c)), NO production in the sham group was 50.7 *μ*mol/L/mg, while NO production in the control group significantly increased to 66.18 *μ*mol/L/mg. Further, the NO production in the ECJM50, ECJM100, and donepezil groups in the kidneys showed no significant difference when compared with the control group. These results indicated that the levels of NO in the brain, liver, and kidney were significantly increased by injection of A*β*_25-35_, and the administration of ECJM and donepezil inhibited NO production in tissues.

### 3.5. Effect of ECJM on Lipid Peroxidation Induced by A*β*_25-35_

**Figure 6:**
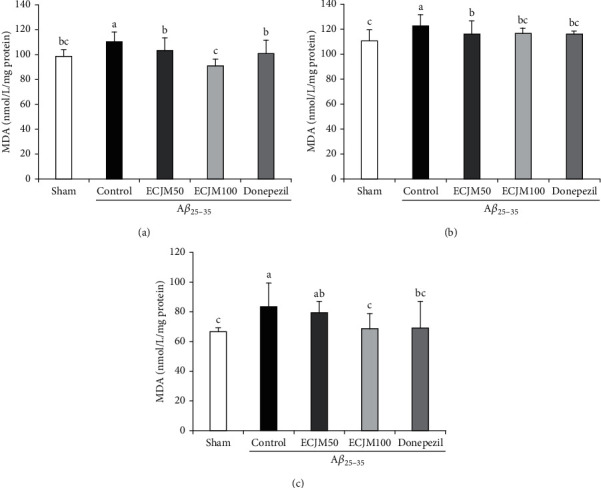
Effect of ECJM on A*β*_25-35_-induced lipid peroxidation in the brain (a), liver (b), and kidney (c). (1) Sham group: injection of 0.9% NaCl and oral administration of water. (2) Control group: injection of A*β*_25-35_ and oral administration of water. (3) ECJM50 group: injection of A*β*_25-35_ and oral administration of ECJM (50 mg/kg/day). (4) ECJM100 group: injection of A*β*_25-35_ and oral administration of ECJM (100 mg/kg/day). (5) Donepezil group: injection of A*β*_25-35_ and oral administration of donepezil (5 mg/kg/day). Values are the mean ± SD. One-way ANOVA was used to compare the mean values of groups, followed by Duncan's multiple-range test post hoc. Different alphabet letters (a, b, and c) indicate statistically significant difference (*P* < 0.05). Mean values with the same letter are not significantly different.

We measured the effect of ECJM on the lipid peroxidation of A*β*_25-35_-induced oxidative stress in the brain, liver, and kidney, and the results are shown in Figure 6. The MDA level in the sham group in the brain (Figure 6(a)) was 98.95 nmol/mg protein, while that in the control group significantly increased to 110.69 nmol/mg protein. Moreover, the MDA levels in the ECJM50, ECJM100, and donepezil groups significantly reduced to 103.84, 91.89, and 101.41 nmol/mg, respectively, compared with the control group. In particular, the MDA level in the ECJM100 group decreased markedly compared to the other groups. In the liver (Figure 6(b)), the MDA level in the sham group was 111.04 nmol/mg protein, while that in the control group increased to 122.71 nmol/mg protein. The MDA levels in ECJM50, ECJM100, and donepezil groups significantly decreased to 116.56 nmol/mg, 116.87 nmol/mg, and 116.35 nmol/mg, respectively, compared with the control group; these results showed that the MDA levels in the ECJM group underwent a similar reduction like the donepezil group. In the kidney (Figure 6(c)), the MDA level in the control group significantly increased to 83.73 nmol/mg protein. However, MDA levels in the ECJM100 and donepezil groups decreased to 68.56 nmol/mg and 69.38 nmol/mg, respectively. The present results indicated that A*β*_25-35_ elevated lipid peroxidation in the brain, liver, and kidney, but oral administration of ECJM attenuated the lipid peroxidation caused by A*β*_25-35_.

## 4. Discussion

AD is characterized by the formation of senile plaques and neurofibrillary tangles in the brain with progressive cognitive impairment due to neuronal death, synaptic dysfunction, and oxidative stress [30]. Oxidative stress induced by the overproduction of ROS, such as hydrogen peroxide, NO, superoxide, and hydroxyl radicals, causes neuronal cell dysfunction or death [31]. Oxidative stress is regulated by homeostasis between oxidants and antioxidants, but overproduction of ROS leads to oxidative modification of proteins, lipids, RNA, DNA, and other biomolecules [32]. The oxidative stress induced by A*β* in the brain is consistent with the injury described in AD [33]. In particular, APP-derived A*β* peptides are inserted into the bilayer of neurons and glial membranes, thereby generating oxygen-dependent free radicals [34]. A*β*_25-35_, a core toxic fragment in A*β* peptide, leads to cognitive deficits, memory impairment, inflammation, and oxidative stress in the mouse brain [35]. Furthermore, previous studies have reported that i.c.v. injection of A*β*_25-35_ is responsible for cognitive impairment in the mouse brain [36]. Therefore, in the present study, we used i.c.v. injection of A*β*_25-35_ to induce cognitive impairment in an AD mouse model.

Donepezil, which is an acetylcholinesterase inhibitor, is a representative drug used for AD treatment by approved the Food and Drug Administration [37]. The concentration of donepezil used in present study was determined on the basis of other reports on animal experiments. Several behavioral studies have proved that donepezil treatment with dose ranging of 3-10 mg/kg prevented the learning and memory deficits by scopolamine treatment [38]. Additionally, previous studies investigated the concentration of donepezil as the most common on 3-5 mg/kg/day by oral administration [39]. Su et al. [40] indicated that oral administration of donepezil (5 mg/kg/day) improved the decreased level of choline acetyltransferase in isoflurane-exposed mice. Therefore, donepezil was treated at 5 mg/kg/day in the present study.

Plant-derived constituents are separated by solvent extraction, and the extract yields and biological activities of plant substances are strongly dependent on the features of the extracting solvent [41]. Solvents frequently used in extraction are polar (e.g., EtOH, methyl alcohol, and water), intermediate polar (e.g., acetone and dichloromethane), and nonpolar (e.g., *n*-hexane and chloroform). EtOH and methyl alcohol have been widely applied to extract all primary (e.g., carbohydrates, organic and amino acids, and vitamins) and secondary metabolites, especially phenolic compounds and flavonoids from various plants [42, 43]. Emerging evidence has shown that ECJM from EtOH extract has relative inhibitory effects against A*β*_25-35_-induced cytotoxicity in neurons and glial cells, as well as against aldose reductase, *α*-glucosidase, oxidative stress, and inflammation [14, 17, 18, 20, 21, 44]. Hence, in the present study, EJCM from EtOH extract was used to investigate the improvement effects of A*β*_25-35_-induced cognitive impairment.

CJM is a medicinal plant with anti-inflammatory, antitumor, antioxidant, and antidiabetic effects [14, 16, 17]. In particular, CJM has been reported to inhibit ROS production and BACE1 activity, indicating that CJM prevents oxidative stress-related diseases such as AD [17, 45]. Additionally, treatment with CJM increases cell viability and reduced ROS production in C6 glial cells induced by A*β*_25-35_ [44]. However, the protective effect of ECJM against cognitive impairment in an A*β*-induced *in vivo* AD mouse model has not been reported. In our current study, ECJM at doses of 50 and 100 mg/kg/day was orally administered to an AD mouse model induced by A*β*_25-35_, and we confirmed its cognitive improvement effects and protective mechanisms against AD in an *in vivo* system.

A*β*-exposed mice can experience severe deficits in spatial memory and learning, but not locomotor and sensorimotor activities [46, 47]. Nga et al. [46] reported that A*β* exposure led to a complete disruption of object recognition and serious detriment in the water maze but did not affect spontaneous locomotor activity or sensorimotor gating in rats. In the present study, we designed behavioral experiments to confirm the recognition, memory, and learning abilities of an A*β*-exposed AD mouse model.

The novel object recognition test is a task based on spontaneous behaviors and is used to evaluate novel object recognition impairment in rodents [48]. When setting a novel object after exposure to familiar objects, rodents spend more time exploring novel objects than familiar objects [49]. We conducted a novel object recognition test to evaluate the protective effect of ECJM on novel object recognition impairment in an A*β*_25-35_-induced mouse model. Our results showed that there was no statistically significant difference between the familiar and novel objects in the A*β*_25-35_-induced control group. Similarly, previous studies demonstrated that A*β*_25-35_-induced cognitive impairment mice did not show statistical significance in differentiating between familiar and novel objects, indicating that the cognitive impairment mouse model was successfully induced in this study. Several studies have reported that AD mice who received oral administration of anti-AD materials such as donepezil, galantamine, and memantine spent more time exploring novel objects [50, 51]. In our study, oral administration of ECJM at a dose of 100 mg/kg/day to AD mice induced a higher awareness of novel objects compared with the control group, indicating that oral administration of ECJM improves novel object recognition in A*β*_25-35_-induced mice.

The T-maze test is widely used to evaluate spatial memory ability [52], and it has been reported that A*β*_25-35_-induction of mice leads to spatial memory deficits. In the present study, the A*β*_25-35_-induced control group showed no significant difference in differentiating between the new and old routes. However, in the spatial exploration of the new and old routes, the ECJM50 and ECJM100 groups had a high exploration rate for the new route compared with the control group. Moreover, the ECJM100 group showed a higher spatial exploration rate than the donepezil-administered group. Therefore, our results indicate that oral administration of ECJM has a protective effect on spatial memory deficits in A*β*_25-35_-induced mice.

To evaluate the long-term memory ability of ECJM in the A*β*_25-35_-induced mouse model, we performed the Morris water maze test. Previous studies have reported that mice induced by A*β*_25-35_ exhibited learning and memory impairment by reducing the time to reach the hidden platform in the Morris water maze [5, 53]. The control group took a longer time to find the hidden escape platform, indicating that the long-term spatial memory of the mice was impaired after A*β*_25-35_ injection. Further, the ECJM50 and ECJM100 groups could quickly find the hidden platform compared with the control group. After removing the hidden platform under the water, the ECJM50 and ECJM100 mice spent more time than the control group in the area where the hidden platform was placed. At the same time, in the experiment of exposing the platform, there was no statistical significance regarding the time for the mice to find the platform among all groups. These results indicate that ECJM can improve long-term spatial memory impairment caused by A*β*_25-35_ in mice, which is related to memory function, but has no effect on swimming and visual abilities.

NO acts as a neurotransmitter that produces oxidants in the brain. An increased level of NO in the brain leads to oxidative stress-mediated cell apoptosis in neurons and is largely involved in the dysfunction of learning and memory [54, 55]. At low concentrations, NO affects the oxidation of the substrate, but NO at high concentrations affects the nitration of proteins and neuronal death, thus suggesting that increased NO concentration plays a neurotoxic role [56]. The overproduction of NO not only exacerbates the inflammatory response but also promotes neurodegeneration [57]. A*β*-mediated neurotoxicity is caused by overproduction of NO, suggesting that production of NO may be a mediator in A*β*-related AD [58]. The reaction of peroxynitrite with protein tyrosine residues resulted in high levels of nitrotyrosine in the brains of AD patients, suggesting that NO participates in the development of AD [59]. NO induced by A*β*_25-35_ releases nitrite (NO_2_^−^) and nitrate (NO_3_^−^) in tissues such as the brain, liver, and kidney. Therefore, the measurements of NO_2_^−^ and NO_3_^−^ concentrations are usually used as indicators of NO production in AD mice [60]. The Griess reaction, which reacts with NO_2_^−^ in mouse tissue homogenates, is widely used as a measurement of the NO_2_^−^ level [61]. Studies have shown that excessive NO production in A*β*-induced mice and neuronal cells induces cognitive impairment and mitochondrial apoptosis [62]. Moreover, an NO-related research demonstrated that inhibitors of NO synthesis attenuated memory deficits in arsenic-induced rodents [63]. Furthermore, a reliable study indicated that the injection of A*β* in the brain of rats damaged spatial learning and memory and produced a high NO concentration [35]. In our study, the protective effects of ECJM on A*β*_25-35_-induced oxidative stress in the mouse brain, liver, and kidney were investigated. Higher NO production was observed in the A*β*_25-35_-induced group in this study. Conversely, the ECJM50 and ECJM100 groups showed significantly decreased NO production in the brain and liver. These results indicate that oral administration of ECJM attenuated oxidative stress by inhibiting NO levels.

Oxygen and polyunsaturated fatty acids (PUFA) are widely found in the lipid bilayer of the brain, and the interaction between PUFA and free radicals leads to lipid peroxidation [64]. Lipid peroxidation and A*β* accumulation have been observed in patients with AD. Additionally, lipid peroxidation leads to A*β* accumulation and plays a role in oxidative stress-induced cognitive impairments, which results from the atrophy and death of neurons in the brain [65]. As reported in previous papers, suppression of lipid peroxidation using antioxidants such as *α*-tocopherol and docosahexaenoic acid is an effective treatment for cognitive dysfunction associated with AD symptoms, including memory and learning ability [66, 67]. Several studies have reported a positive correlation between NO and lipid peroxidation. In previous studies using animal models of AD, NO and lipid peroxidation can cause A*β* accumulation during the progression of AD [68].

MDA is a product of peroxidation of PUFAs, and the level of MDA indicates the level of lipid peroxidation [69]. Studies have reported an increase in MDA levels in the AD brain, and this has been considered a biomarker of lipid peroxidation [70]. Our results showed that MDA levels in the A*β*_25-35_-induced control group were significantly increased in the brain, liver, and kidney, compared with those in the sham group. Furthermore, the ECJM50 and ECJM100 groups showed significantly decreased MDA levels in the brain and liver tissues, compared with the control group. Therefore, administration of ECJM inhibited oxidative stress by reducing lipid peroxidation in A*β*_25-35_-induced AD mouse.

## 5. Conclusions

The results of the present study showed that oral administration of ECJM (50 mg/kg/day and 100 mg/kg/day) improved cognitive abilities by inhibiting oxidative stress in A*β*_25-35_-induced mice. This study suggests that CJM could be useful as an agent for the prevention and treatment of AD.

## Data Availability

The data used to support the findings of this study are included within the article.
